# Surface Electromyography Thresholds as a Measure for Performance Fatigability During Incremental Cycling in Patients With Neuromuscular Disorders

**DOI:** 10.3389/fphys.2022.821584

**Published:** 2022-03-17

**Authors:** Nicoline B. M. Voet, Christiaan G. J. Saris, Dick H. J. Thijssen, Vincent Bastiaans, David E. Sluijs, Mariska M. H. P. Janssen

**Affiliations:** ^1^Department of Rehabilitation, Radboud University Medical Center, Donders Institute for Brain, Cognition and Behaviour, Nijmegen, Netherlands; ^2^Klimmendaal, Rehabilitation Center, Arnhem, Netherlands; ^3^Department of Neurology, Radboud University Medical Center, Donders Institute for Brain, Cognition and Behaviour, Nijmegen, Netherlands; ^4^Department of Physiology, Radboud University Medical Center, Donders Institute for Brain, Cognition and Behaviour, Nijmegen, Netherlands; ^5^Sports Medicine Center, HAN Seneca, HAN University of Applied Sciences, Nijmegen, Netherlands

**Keywords:** muscle fatigue, neuromuscular disorders, exercise, performance fatigability, cycling, surface electromyography thresholds, ventilatory thresholds, leg muscles

## Abstract

In healthy persons, there is an excellent relation between the timing of the (two) surface electromyography (sEMG) thresholds and the (two) ventilatory thresholds during exercise. The primary aim of this study was to determine the relative timing of both sEMG and ventilatory thresholds in patients with neuromuscular disorders compared with healthy subjects during a maximal ergospirometry cycling test. We hypothesized that in patients with neuromuscular disorders, the sEMG thresholds would occur relatively earlier in time than the ventilatory thresholds, compared to healthy subjects, because performance fatigability occurs more rapidly. In total, 24 healthy controls and 32 patients with a neuromuscular disorder performed a cardiopulmonary exercise test on a bicycle using a 10-min ramp protocol, during which we collected ergospirometry data: power at both ventilatory and sEMG thresholds, and sEMG data of lower leg muscles. In line with our hypothesis, normalized values for all thresholds were lower for patients than healthy subjects. These differences were significant for the first ventilatory (*p* = 0.008) and sEMG threshold (*p* < 0.001) but not for the second sEMG (*p* = 0.053) and ventilatory threshold (*p* = 0.238). Most parameters for test–retest reliability of all thresholds did not show any fixed bias, except for the second ventilatory threshold. The feasibility of the sEMG thresholds was lower than the ventilatory thresholds, particularly of the first sEMG threshold. As expected, the sEMG thresholds, particularly the first threshold, occurred relatively earlier in time than the ventilatory thresholds in patients compared with healthy subjects. A possible explanation could be (a combination of) a difference in fiber type composition, disuse, and limited muscle-specific force in patients with neuromuscular disorders. sEMG measurements during submaximal dynamic exercises are needed to generalize the measurements to daily life activities for future use in prescribing and evaluating rehabilitation interventions.

## Introduction

Approximately 1 in every 625 persons worldwide experiences some type of neuromuscular disorder (NMD) in their lifetime (Mary et al., 2018). A NMD is a disease that affects any part of the neuromuscular pathway that starts with the motor cortex, and ends in the muscle, going through the upper and lower motor neurons (Feasson et al., 2006). People with NMD are subject to all types of fatigue. One of the most important distinctions in a discussion of fatigue is that between perception of fatigue, often described as the sensation of discomfort and lack of energy caused by exercise or clinical conditions (Twomey et al., 2017), and performance fatigability, defined as the failure to maintain the required or expected force in a single muscle or muscle group, leading to a reduced performance (Asmussen, 1979; McDonald, 2002).

In healthy adults, perceptions of fatigability are predictable and transient phenomena typically brought about by prolonged exertion that diminish with rest and do not interfere with usual daily activities. Establishing an association between fatigability and fatigue complaints is an important goal for clinical research but has proven difficult for most conditions (Kluger et al., 2013). Perceptions of fatigue and fatigability are potentially independent in chronic neurological disorders. In Parkinson’s disease, for example, Lou et al. (2003) found objective decrements in performance fatigability did not significantly correlate with perceived fatigue. Similarly, in multiple sclerosis, changes in performance fatigability during prolonged exercise testing can occur independently of changes in perceived fatigue (Bailey et al., 2007). Patients with NMD do not always detect fatigability in real-time. As a result, for patients, it is difficult to cope with performance fatigability in daily life, which often leads to overuse and, in the end, a vicious circle of inactivity and a further decline in (muscle) performance. Valid measures for fatigability could help people with NMD find the right intensity of daily life activities and exercise. Performance fatigability is primarily measured by quantifying the decline in performance during a prolonged physical task. For properly assessing performance fatigability, it is critical to use a valid task as well as a valid measure (Kluger et al., 2013). In patients with NMD, muscle weakness and performance fatigability are more often a limiting factor than cardiorespiratory fitness during daily life activities.

Moreover, the majority of the patients with NMD are bothered more by performance fatigability than they are by muscle weakness, as performance fatigability highly fluctuates and is generally less predictable than muscle weakness. Although performance fatigability is a common and potentially the most prominent and disabling symptom of NMD (Kalkman et al., 2007), there is still much unknown about its mechanism and possible treatment options (Voet, 2019). Previous research described that surface electromyography (sEMG) has an excellent reliability for assessing signs of performance fatigability during a cycle test in healthy subjects (Singh et al., 2007; Hug and Dorel, 2009). sEMG reflects both central and peripheral neuromuscular properties since its main characteristics, such as amplitude and power spectrum, depend on muscle fiber membrane properties and the timing of motor unit action potentials (Farina et al., 2014). An increase in sEMG amplitude reflects an increase in motor unit recruitment and modulation of their discharge rates to compensate for the deficit in contractility caused by fatigued motor units (Moritani and deVries, 1978). Several studies in healthy participants have reported a deviation from linearity in the relationship between the Root Mean Square (RMS), the amplitude of sEMG, and the workload level during incremental cycle-ergometer exercise (Iannetta et al., 2017). This breakpoint is referred to as the sEMG threshold (sEMG Th). Although several explanations for its occurrence have been formulated, the scientific community still has not reached a consensus for its physiological cause (Ertl et al., 2016). Ertl et al. (2016) concluded in their review that, in general, it appears that sEMG Ths computed with the amplitude-based methods (RMS, iEMG) are more consistent than techniques based on the frequency using the mean power frequency or median frequency. Researchers suggest that the sEMG Th is attributable to an increased contribution of fast-twitch motor units to maintain the required energy supply for muscle contraction and compensate for the loss of contractility (Moritani and deVries, 1978; Jurimae et al., 2007; Enoka and Duchateau, 2008; Briscoe et al., 2014; Ertl et al., 2016). The increased contribution of fast-twitch motor units results in a faster fatigability of the involved muscles because of the increased H^+^ and lactate concentrations. Furthermore, Candotti et al. (2008) reported a correlation between the percentage of fast-twitch muscle fiber recruitment and the lactate accumulation after the sEMG Th.

So far, there is an excellent relation between the timing of the (two) sEMG Ths and the (two) ventilatory thresholds (VTs) during exercise in healthy controls (HC) (Moritani and deVries, 1978; Lucia et al., 1997; Chwalbinska-Moneta et al., 1998; Hug et al., 2003; Tikkanen et al., 2012; Ertl et al., 2016). The first VT (VT1), or respiratory compensation point, as measured during a maximal cycling test, using breath-by-breath gas analysis, refers to the point at which arterial partial pressure of carbon dioxide starts to decline during heavy exercise (Wasserman, 1978). It has been interpreted as a ventilatory response to lactic acidosis. At the second VT (VT2), hydrogen ions released from lactic acid can no longer be buffered by blood bicarbonate stores, and thus, metabolic acidosis results. This increase in hydrogen ions further stimulates ventilation by stimulating central and peripheral chemoreceptors, resulting in a further increase in ventilation (Wasserman et al., 1990). As VTs produce all muscles’ overall systemic response, detecting sEMG Ths in specific muscles would probably be more suitable for NMD patients as muscle weakness can be focal and asymmetrical (Wasserman et al., 1990). However, there has never been any study about the measurement of sEMG Ths in patients with NMD, as far as we know.

The primary aim of this study was to determine the relative timing of both sEMG Ths and the VTs in patients with NMD compared with HC during a maximal cycling test. To do so, we aim to study the relative difference in timing between the sEMG Ths and VTs in patients with NMD and compare the findings with HC. We expect to find significantly lower normalized values for sEMG Ths and VTs in NMD patients than HC. Moreover, since in patients with NMD, compared to HC, performance fatigability is more frequently a limiting factor in daily life activities than cardiorespiratory fitness, we hypothesized the sEMG Ths to occur at relatively and absolute lower power values than the VTs in patients with NMD, compared to the HC. The secondary aims of this study were to determine and compare the feasibility and reliability of the sEMG Ths and VTs in NMD patients. We expect that the reliability of the sEMG Ths in both HC and patients will be higher than the reliability of the VTs, as the sEMG Ths are more muscle-specific and the VTs are a derivative from the muscular and metabolic system, provided that cardiorespiratory functioning is not impaired.

## Materials and Methods

### Participant Characteristics

**FIGURE 1:**
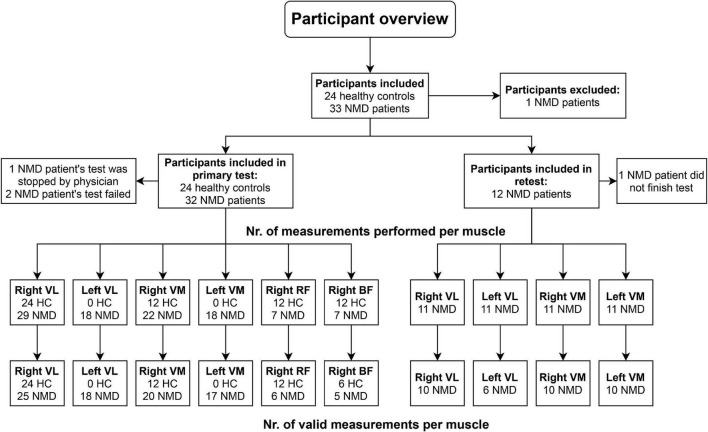
Overview of measurements performed in the study. HC, healthy controls; NMD, patients with neuromuscular disorders; VL, vastus lateralis; VM, vastus medialis; RF, rectus femoris; BF, biceps femoris.

Healthy participants were recruited through advertisements on social media, and NMD patients were recruited through the Radboud University Medical Center (Radboudumc) outpatient clinic. All patients were diagnosed with a slowly progressive NMD on clinical and genetic grounds. Participants were excluded from the study if they had cognitive impairments, used beta-blockers medication, could not cycle for 20 min, or had contraindications to conduct a maximal cardiopulmonary stress test according to the AHA guidelines (Gibbons et al., 2002). This study was approved by the medical ethical committee Arnhem–Nijmegen, the Netherlands (Registration number 2019-5467). Informed consent was obtained from all participants. Figure 1 gives a graphical overview of the participants included in this study and the number of available measurements per muscle.

### Procedure

All participants performed a maximal incremental ergospirometry cycle test using a steep ramp protocol. The test was performed on an electromagnetically braked leg cycle ergometer (Excalibur Sport 2006, Lode, Groningen, Netherlands), which was connected to the Quarck Cardio Pulmonary Exercise Test (CPET) (Cosmed, Rome, Italy).

The exercise protocol consisted of a 1-min resting phase, a 3-min warming-up at 0 W, 3-min warming-up at 10–15% of expected power output, a ramp protocol in which workload gradually increased with 5–35 W per minute (based on expected power), and a cooling down. The power increase in the ramp protocol was estimated based on gender, age, fitness level, and the Medical Research Council (MRC) score of a participant. This muscle scale grades muscle power on a scale of 0–5 in relation to the maximum expected for that muscle. The maximum performance was aimed to be achieved within 8–12 min. Patients were instructed to cycle with a pedal frequency above 60 rpm, the progressive loading phase was terminated, and a 3-min recovery phase was initiated/commenced until exhaustion. When the pedal frequency dropped below 60 rpm, the test was terminated. Participants’ feet were strapped to the pedals of the Excalibur bike. A medical doctor was present for the whole duration of the test. We performed a breath-by-breath analysis of pulmonary gas exchange using this setup, including continuous 12-channel ECG monitoring and blood pressure measurement at multiple moments throughout the test. In addition, we simultaneously measured the sEMG of several leg muscles with the TMSi Mobi (TMSi, Oldenzaal, Netherlands). This paper combines the results of multiple smaller studies in both HC and NMD patients.

### Outcome Measures

#### Ergospirometry

The Metasoft Studio system (Cortex, Leipzig, Germany) was used for breath-to-breath gas analysis. The minute ventilation, oxygen consumption, and carbon dioxide production were collected. The power (W) at the VT1 and the VT2 was determined with this data. The first breakpoint is VT1; the second breakpoint is VT2. VT1 and VT2 were visually detected as two break points in the linearity of the minute ventilation by two experienced independent examiners. Several methods were combined to determine the VT1 and VT2; the V-slope method, the ventilatory equivalent method, and the PET method. The V-slope method of determining the anaerobic threshold makes use of the fact that carbon dioxide production (VCO_2_) plotted against oxygen consumption (VO_2_) shows a slope of slightly less than one for work below the anaerobic threshold (Wasserman et al., 1990; Graham et al., 2019).

#### Surface Electromyography

**FIGURE 2:**
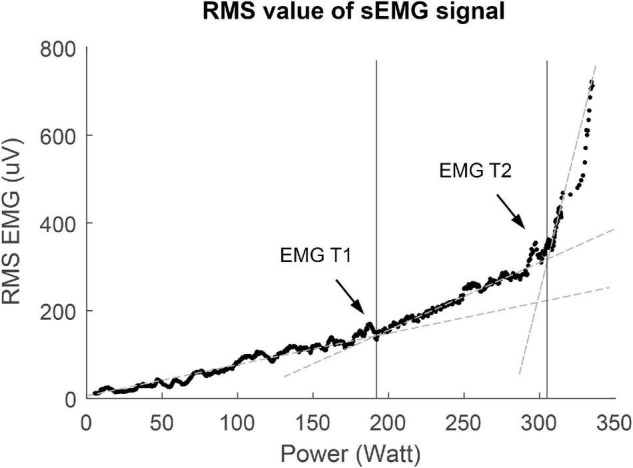
Example of V-slope method used for threshold determination of the first surface EMG threshold (sEMG Th1) and the second surface EMG threshold (sEMG Th2). RMS, root mean square.

sEMG signals of maximal four different leg muscles [vastus lateralis (VL), vastus medialis (VM), rectus femoris (RF), biceps femoris (BF)] per participant were collected during the cycling test (see Figure 1). These muscles were chosen because the quadriceps and hamstring muscles are important during cycling, and literature has shown that sEMG Ths could be detected in these muscles (Lucia et al., 1999; Hug et al., 2006). Disk-shaped Ag-AgCL ARBO ECG electrodes (Kendall™, Cardinal Health, Dublin, Ireland) with a diameter of 24 mm were placed at an inter-electrode distance of 24 mm after the skin was shaven or scrubbed clean. Electrodes were placed according to the SENIAM guidelines (Hermens et al., 2000). sEMG data were collected with a sampling frequency of 2,048 Hz. The raw sEMG data were filtered in Matlab using a bandpass filter (20–450 Hz) to remove movement artifacts. Moving averages of the RMS of the sEMG signals were calculated over a window size of 10 s, which step size of 1 s. The sEMG Ths were visually detected from the RMS graphs (Figure 2). The power (W) at the first breakpoint in the graph (if present) was identified as sEMG Th1, the power at the second breakpoint was identified as sEMG Th2. Two researchers (NV and MJ) determined the Ths independently for inter-rater reliability analysis. When there was a difference of more than 5 W between the two researchers, the specific Ths were analyzed again until consensus over the Ths was reached.

### Data Analysis

Feasibility of both the maximal incremental ergospirometry cycle test and the determination of the VTs and sEMG Ths was determined by the percentage of participants that could complete the exercise test and the percentage of participants in which the different thresholds (VT1, VT2, sEMG Th1, and sEMG Th2) could be determined. The maximal incremental ergospirometry cycle test and the determination of the VTs and sEMG Ths were found feasible when they could be, respectively, performed and determined in >75% of the participants.

### Statistics

The first step of the reliability analysis was to determine the inter-rater reliability of the power at sEMG Th1 and sEMG Th2 by calculating the intraclass correlation coefficient (ICC) and mean difference between the values scored by examiner one compared to the scores of examiner two. The second step of the reliability analysis was to determine the test–retest reliability for maximal power (Pmax) and power at VT1, VT2, sEMG Th1, sEMG Th2 of the first test compared to the second test.

To gain insight into the relationship between the VTs and the sEMG Ths, we calculated the mean difference and limits of agreement between VT1 and sEMG Th1 and between VT2 and sEMG Th2. In addition, we determined the construct validity using student *t*-tests on the difference between NMD patients and HC. To make a relative comparison between patients and HC possible, we normalized the occurrence of both ventilatory and sEMG Ths by calculating the power at the thresholds as a percentage of the maximal power cycled.

The ICC values were calculated using a two-way mixed model with absolute agreement [ICC (3,1)]. Inter-rater reliability was acceptable if ICC > 0.7, good if ICC > 0.8, and excellent if ICC > 0.9 (Kottner et al., 2011). Test–retest reliability and the relation between VTs and sEMG Ths was assessed using a Bland-Altman analysis to determine mean bias and limits of agreement [95%-confidence intervals (CI)] of the variables. In addition, linear regression analyses were performed to examine proportional bias in the data. Student *t*-tests were used to test for statistical differences between HC and NMD patients. The mean group differences with 95% confidence intervals were calculated.

All statistical analyses were performed using IBM SPSS statistics 25.0 for Windows (SPSS Inc., Chicago, IL, United States).

## Results

### Participant Characteristics

Table 1 shows an overview of the participant characteristics. The percentage of males in the NMD patient group (59%) was higher compared to the HC group (33%). In addition, the average age of the NMD patient group was significantly higher than the HC group (*p*-value < 0.001). The NMD patient group consisted of a large range of conditions: bulbospinal muscular atrophy (*n* = 1), cardiomyopathy with involvement of muscle tissue (*n* = 1), chronic idiopathic axonal polyneuropathy (*n* = 2), chronic progressive external ophthalmoplegia (*n* = 1), myotonic dystrophy type 1 (*n* = 4), facioscapulohumeral muscular dystrophy (*n* = 13), Charcot Marie tooth (*n* = 4), myasthenia gravis (*n* = 1), myopathy (*n* = 2), necrotizing autoimmune myopathy (*n* = 1), poliomyelitis (*n* = 1), and sarcoid myopathy (*n* = 1). For the retest, patients bulbospinal muscular atrophy (*n* = 1), cardiomyopathy with involvement of muscle tissue (*n* = 1), chronic progressive external ophthalmoplegia (*n* = 1), myotonic dystrophy type 1 (*n* = 2), facioscapulohumeral muscular dystrophy (*n* = 3), poliomyelitis (*n* = 1), Charcot Marie tooth (*n* = 2), and necrotizing autoimmune myopathy (*n* = 1) participated in the study.

**TABLE 1:** Participant characteristics.

Healthy controls/NMD patients	24	32
Nr. of participants		
Gender (nr. male/nr. female)	8/16	19/13
Age (years) (mean, range)	36.4 (19.1 – 59.6)	55.0 (30.1 – 75.0)
Height (cm) (mean, range)	176.6 (165.0 – 191.0)	176.8 (157.0 – 198.0)
Weight (kg) (mean, range)	69.6 (56.0 – 101.0)	81.2 (51.4 – 114.0)
BMI (mean, range)	22.3 (18.5 – 35.0)	25.9 (19.4 – 37.4)

*NMD, neuromuscular disorder; BMI, body mass index.*

### Feasibility

In total, 56 (24 HC and 32 NMD patients) people performed an exercise test. In addition, 12 NMD patients performed two tests for the test–retest reliability. Three NMD patients were excluded from analysis as they did not complete the exercise test (1 test was stopped by the physician present at the measurement, two tests, and one retest were stopped prematurely by the participants). The other participants all completed the exercise test, however, not all data could be used for analysis. For the HC, sEMG signals of the biceps femoris were not recorded correctly in six out of 12 participants due to a damaged sEMG cable. For the primary exercise test in NMD patients, data of 18 out of 145 (12.4%) measured muscles contained a measurement error, either due to a faulty cable, disturbance in sEMG signal, or human error.

Table 2 shows an overview of the percentage of thresholds that could be determined for both VTs and sEMG Ths. For HC, sEMG Th1 could best be determined for the right BF muscle (66.7%) and worst for the right VL (41.7%). For sEMG Th2, the highest percentage of thresholds could be determined for the right RF (100%), and the lowest percentage thresholds could be determined in the right BF (83.3%). For NMD patients, sEMG Th1 could best be determined for the VM left (37.0%) and worst for the right RF (16.7%). For sEMG Th2, the highest percentage of thresholds could be determined for the VL right (91.4%), and the lowest percentage thresholds could be determined in the right BF (80.0%).

**TABLE 2:** Percentage of thresholds that could be determined.

		Healthy controls		NMD patients (test and retest combined)	
		*N*-measured	Percentage of thresholds determined (%)	*N*-measured	Percentage of thresholds determined (%)
**VT1**		24	95.8	40	100
**VT2**		24	95.8	40	96.6
**sEMG Th1**					
	Right VL	24	41.7	35	22.9
	Left VL	0	n.a.	24	29.2
	Right VM	12	50.0	30	36.7
	Left VM	0	n.a.	27	37.0
	Right RF	12	50.0	6	16.7
	Right BF	6	66.7	5	20.0
	**All muscles**	54	52.1	127	27.1
**sEMG Th2**					
	Right VL	24	87.5	35	91.4
	Left VL	0	n.a.	24	83.3
	Right VM	12	91.7	30	86.7
	Left VM	0	n.a.	27	88.9
	Right RF	12	100.0	6	83.3
	Right BF	6	83.3	5	80.0
	**All muscles**	54	90.6	127	85.6

*NMD, neuromuscular disorder; VT1, first ventilatory threshold; VT2, second ventilatory threshold; VL, vastus lateralis; VM, vastus medialis; RF, rectus femoris; BF, biceps femoris.*

### Inter-Rater Reliability

Two examiners independently determined the sEMG Ths for all valid measurements (Table 3, nr. observations). The percentage of thresholds scored represents the percentage of observations in which an examiner could determine a threshold. N-valid represents the number of observations that both examiners scored. Both examiners’ average percentage of valid measurements is 7.7% for sEMG Th1 and 77.6% for sEMG Th2. Table 2 also shows the ICC values for inter-rater reliability. For sEMG Th1, ICC values could only be calculated in three muscles (right VL, right VM, and right RF) due to the small number of observations. For sEMG Th2, ICC values could be calculated for all muscles and were high (ICC > 0.9). There does not appear to be significant restriction of range or gross violations of normality based on the Shapiro–Wilk test for normality and inspection of the histograms.

**TABLE 3:** Inter-rater reliability for both sEMG thresholds for all muscles, healthy controls and patients combined.

Threshold – muscle	Nr. observations	% of thresholds scored by Examiner 1	% of thresholds scored by Examiner 2	*N*-valid	Examiner 1 Power (W) [mean (*SD*)]	Examiner 2 Power (W) [mean (*SD*)]	ICC (95% CI)
sEMG Th1 – Right VL	59	33.9	15.3	5	247.8 (57.1)	201.6 (39.5)	0.169 (−1.848; 0.894)
sEMG Th1 – Left VL^†^	24	12.0	8.0	1	n.a.	n.a.	n.a.
sEMG Th1 – Right VM	42	38.1	19.0	4	239.5 (49.6)	181.8 (31.1)	0.358 (−0.509; 0.935)
sEMG Th1 – Left VM^†^	27	14.8	22.2	0	n.a.	n.a.	n.a.
sEMG Th1 – Right RF	18	44.4	33.3	3	188.3 (127.8)	173.7 (116.1)	n.a.
sEMG Th1 – Right BF	11	27.3	9.1	1	n.a.	n.a.	n.a.
sEMG Th2 – Right VL	59	86.4	89.8	49	190.7 (83.9)	185.6 (79.9)	0.990 (0.981; 0.995)*
sEMG Th2 – Left VL^†^	24	96.0	80.0	20	142.7 (53.0)	136.3 (50.2)	0.955 (0.887; 0.982)*
sEMG Th2 – Right VM	42	92.9	92.9	38	177.3 (81.9)	172.8 (79.1)	0.991 (0.983; 0.996)*
sEMG Th2 – Left VM^†^	27	92.6	77.8	21	141.6 (51.7)	135.9 (47.8)	0.954 (0.888; 0.981)*
sEMG Th2 – Right RF	18	88.9	88.9	15	229.9 (77.9)	213.3 (78.0)	0.964 (0.852; 0.989)*
sEMG Th2 – Right BF	11	100.0	90.9	9	202.7 (98.0)	175.3 (81.6)	0.965 (0.416; 0.994)*

*sEMG Th1, first surface EMG threshold; sEMG Th2, second surface EMG threshold; VL, vastus lateralis; VM, vastus medialis; RF, rectus femoris; BF, biceps femoris.*

^†^Left VL and left VM are only measured in NMD patients and not in healthy controls. *Statistically significant p-value < 0.05.

### Test–Retest Reliability

**FIGURE 3:**
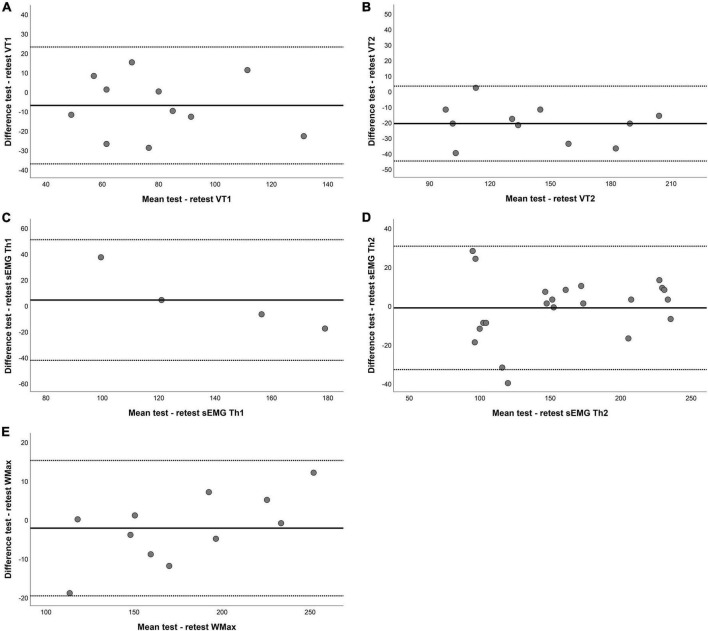
Bland–Altman plots for test–retest reliability of **(A)** the first ventilatory threshold (VT1), **(B)** the second ventilatory threshold VT2, **(C)** the first surface EMG threshold (sEMG Th1), **(D)** the second surface EMG threshold (sEMG Th2), and **(E)** maximum power (Pmax). The horizontal lines represent the mean difference (bias) and two standard deviations, i.e., limits of agreement of the differences between the test and retest.

Twelve patients performed the steep ramp protocol two times to determine test–retest reliability. Table 4 shows the results for Bland-Altman analysis for both VTs and sEMG thresholds. Most parameters did not show any fixed bias, except for VT2. In addition, Pmax showed proportional bias indicating that participants with a larger Pmax also have wider limits of agreement. Figure 3 shows the Bland-Altman plot for VT1, VT2, sEMG Th1 sEMG Th2, and Pmax.

**TABLE 4:** Test–retest reliability for maximal power ventilatory and EMG thresholds of the first test compared to the second test.

Variable		*N*-valid	Test		Retest	Mean difference (LOA)	95% CI	Fixed Bias	Regression	*p*-value	Proportional bias
			Power (W) [mean (SD)]		Power (W) [mean (SD)]						
VT1		11	76.0 (24.9)		83.2 (26.7)	−7.2 (−37.3 to 22.9)	−17.5 to 3.1	No	–0.073	0.732	No
VT2		11	131.4 (37.0)		152.4 (39.3)	−21.0 (−45.1 to 3.1)	−29.3 to −12.7	Yes	–0.062	0.576	No
Pmax		11	177.0 (49.0)		179.3 (43.3)	−2.3 (−19.7 to 15.2)	−8.2 to 3.7	No	0.126	0.030	Yes
sEMG Th1^†^		4	141.0 (24.8)		137.0 (46.9)	4.0 (−42.6 to 50.6)	−33.8 to 41.8	No	–0.627	0.062	No
sEMG Th2^†^		22	158.7 (54.1)		160.0 (50.7)	−1.3 (−33.0 to 30.5)	−8.5 to 5.9	No	0.067	0.342	No
	Right VL	7	165.8 (55.9)	153.4 (48.0)	0.9 (−34.1 to 35.8)	−15.7 to 17.4	No		0.099	0.498	No
	Left VL	4	161.9 (53.5)	152.0 (48.4)	−0.8 (−25.5 to 24.0)	−20.8 to 19.3	No		0.159	0.284	No
	Right VM	5	151.9 (50.0)	161.0 (54.8)	6.0 (−21.3 to 33.3)	−11.3 to 23.3	No		–0.031	0.831	No
	Left VM	6	157.0 (49.3)	158.8 (49.6)	−10.2 (−45.0 to 24.6)	−28.8 to 8.5	No		0.077	0.670	No

*Power at which thresholds are reached.*

*VT1, first ventilatory threshold; VT2, second ventilatory threshold; P, power; sEMG Th1, first surface EMG threshold; sEMG Th2, second surface EMG threshold; LOA, limits of agreement; VL, vastus lateralis; VM, vastus medialis.*

*^†^sEMG Ths combined for all muscles (vastus medialis and vastus lateralis left and right) were used for analysis.*

### Relation Between Ventilatory and Surface Electromyography Thresholds

**FIGURE 4:**
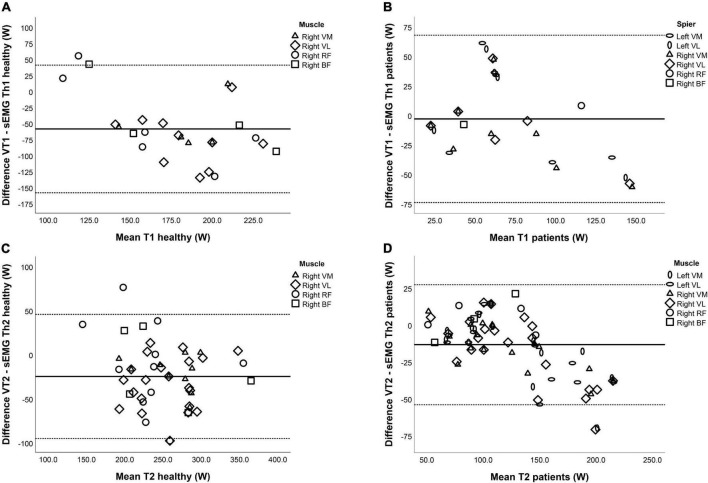
Bland–Altman plots for the relation between the ventilatory thresholds (VTs) and surface EMG thresholds (sEMG Ths) of **(A)** Th1 in healthy controls, **(B)** Th1 in neuromuscular disorder (NMD) patients, **(C)** Th2 in healthy controls, and **(D)** Th2 in NMD patients. The horizontal lines represent the mean difference (bias) and two standard deviations, i.e., limits of agreement of the differences between the VTs and sEMG Ths.

Table 5 shows the mean difference and limits of agreement (LOA) between the VTs and the sEMG Ths for both NMD patients and HC. In NMD patients, there was no fixed bias and no proportional bias between VT1 and sEMG Th1 except for a significant proportional bias when all muscles were combined. The relation between VT2 and sEMG Th2 in NMD patients shows both fixed bias and proportional bias in the left VM, right VM, left VL, and right VL and when looking at all muscles combined. For HC, there is a fixed bias for both Ths for the right VL and right VM muscles; however, there is no proportional bias. In addition, the right RF and right BF muscles did not show any fixed or proportional bias. There was no significant difference for the right RF and right BF. Figure 4 shows the Bland-Altman plots for the difference between the first and second threshold for both NMD patients and HC.

**TABLE 5:** Analysis of the relation between the ventilatory and the sEMG thresholds.

	Muscle	*N*-valid	Mean difference (LOA)	95% CI	Fixed bias	Regression	*p*-value	Proportional bias
**NMD patients threshold 1**								
	Right VL	6	−7.0 (−74.5 to 60.5)	(−43.1 to 29.1)	No	−0.481	0.207	No
	Left VL	5	11.6 (−74.7 to 97.9)	(−43.1 to 66.3)	No	−0.584	0.302	No
	Right VM	8	−15.8 (−79.5 to 48.0)	(−43.0 to 11.5)	No	−0.459	0.134	No
	Left VM	6	3.8 (−83.8 to 91.5)	(−43.1 to 50.8)	No	−0.593	0.336	No
	Right RF	1	n.a.	n.a.	n.a.	n.a.	n.a.	n.a.
	Right BF	1	n.a.	n.a.	n.a.	n.a.	n.a.	n.a.
	All muscles	27	−3.2 (−74.1 to 67.7)	(−17.5 to 11.1)	No	−0.453	0.011	Yes
**NMD patients threshold 2**								
	Right VL	22	−17.6 (−63.3 to 28.1)	(−27.9 to −7.2)	Yes	−0.362	<0.001	Yes
	Left VL	13	−14.6 (−57.0 to 27.8)	(−27.1 to −2.1)	Yes	−0.332	0.004	Yes
	Right VM	18	−13.7 (−46.2 to 18.8)	(−22.0 to −5.5)	Yes	−0.262	0.001	Yes
	Left VM	14	−14.8 (−57.7 to 28.1)	(−27.4 to −2.2)	Yes	−0.325	0.003	Yes
	Right RF	4	4.3 (−14.2 to 22.7)	(−10.8 to 19.3)	No	−0.045	0.783	No
	Right BF	3	4.3 (−28.0 to 36.7)	(−36.7 to 45.3)	No	0.462	0.003	Yes
	All muscles	75	−13.5 (−54.2 to 27.1)	(−18.3 to−8.8)	Yes	−0.3	<0.001	Yes
**Healthy controls threshold 1**								
	Right VL	10	−74.4 (−157.6 to 8.8)	(−104.8 to −44.0)	Yes	−0.162	0.778	No
	Left VL	n.a.	n.a.	n.a.	n.a.	n.a.	n.a.	n.a.
	Right VM	5	−55.6 (−131.2 to 20.0)	(−103.5 to −7.7)	Yes	0.514	0.576	No
	Left VM	n.a.	n.a.	n.a.	n.a.	n.a.	n.a.	n.a.
	Right RF	6	−47.0 (−185.2 to 91.2)	(−121.0 to 27.0)	No	−1.228	0.058	No
	Right BF	4	−42.8 (−158.5 to 73.0)	(−136.7 to 51.2)	No	−0.863	0.218	No
	All muscles	25	−59.0 (−158.5 to 40.5)	(−80.0 to−38.0)	Yes	−0.717	0.010	Yes
**Healthy controls threshold 2**								
	Right VL	21	−32.8 (−91.7 to 26.2)	(−46.5 to −19.1)	Yes	0.111	0.521	No
	Left VL	n.a.	n.a.	n.a.	n.a.	n.a.	n.a.	n.a.
	Right VM	10	−20.3 (−59.2 to 18.6)	(−34.5 to −6.1)	Yes	−0.142	0.470	No
	Left VM	n.a.	n.a.	n.a.	n.a.	n.a.	n.a.	n.a.
	Right RF	12	−19.3 (−120.2 to 81.7)	(−52.0 to 13.5)	No	−0.349	0.270	No
	Right BF	5	−16.4 (−102.3 to 69.5)	(−70.8 to 38.0)	No	−0.280	0.454	No
	All muscles	48	−25.1 (−95.5 to 45.4)	(−35.5 to−14.6)	Yes	−0.2	0.199	No

*NMD, neuromuscular disorder; VL, vastus lateralis; VM, vastus medialis; RF, rectus femoris; BF, biceps femoris.*

**FIGURE 5:**
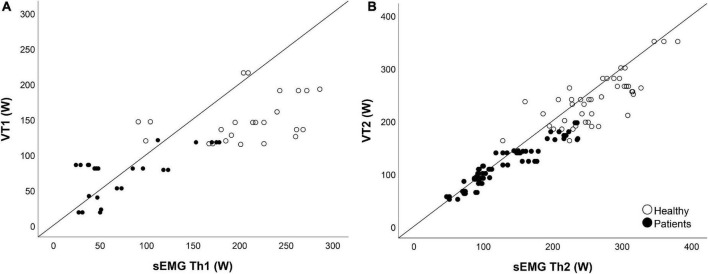
Validity. Panel **(A)** shows the scatterplot of the first ventilatory threshold (VT1) compared to the first surface EMG threshold (sEMG Th1). Panel **(B)** shows the scatterplot of the second ventilatory threshold VT2 compared to the second surface EMG threshold (sEMG Th2). Open circles represent the healthy controls, closed circles represent the neuromuscular disorder (NMD) patients. For the sEMG Ths, all muscles are combined, so data of six muscles are shown.

To test the construct validity, the hypothesis was that the VTs and the sEMG Ths of HC were higher than NMD patients. This was indeed the case for all thresholds (Table 6 and Figure 5). Both the VTs and the sEMG Ths can differentiate between HC and patients, as HC had thresholds at higher power (*p* < 0.001). However, when looking at the normalized thresholds (percentage of maximal power obtained during the endurance test), we saw smaller differences between HC and NMD patients (Table 5). We hypothesized that the sEMG Ths of NMD patients would occur relatively earlier than controls. This was, however, only true for sEMG Th1 (*p* < 0.001) and VT1 (*p* = 0.008), and not for VT2 (*p* = 0.238) and sEMG Th2 (*p* = 0.053).

**TABLE 6:** Construct validity.

Outcome absolute		*N*-valid healthy controls	Healthy controls Power (W) [mean (*SD*)]	*N*-valid NMD patients	NMD patients Power (W) [mean (*SD*)]	*p*-value
**VT1**		23	144.9 (36.7)	30	61.4 (30.4)	<0.001
**VT2**		23	238.0 (42.9)	29	111.2 (39.4)	<0.001
**Wmax**		23	297.0 (42.1)	30	147.9 (55.5)	<0.001
sEMG Th1*						
	Right VL	10	222.4 (35.8)	6	72.5 (55.1)	<0.001
	Left VL	n.a.	n.a.	5	64.4 (59.6)	n.a.
	Right VM	6	207.0 (26.0)	8	77.4 (52.1)	<0.001
	Left VM	n.a.	n.a.	6	72.5 (50.8)	n.a.
	Right RF	6	185.5 (76.8)	1	112.0 ()	n.a.^†^
	Right BF	4	204.5 (78.8)	1	47.0 ()	n.a. ^†^
	**All muscles**	26	207.6 (52.2)	27	73.0 (49.6)	<0.001
sEMG Th2*						
	Right VL	21	271.2 (40.8)	23	140.0 (55.9)	<0.001
	Left VL	n.a.	n.a.	14	131.8 (55.4)	n.a.
	Right VM	11	270.9 (38.1)	18	126.8 (51.6)	<0.001
	Left VM	n.a.	n.a.	14	133.1 (57.1)	n.a.
	Right RF	12	246.3 (64.8)	5	117.0 (55.0)	0.001
	Right BF	5	263.8 (81.6)	4	114.5 (53.3)	0.016
	**All muscles**	49	264.3 (51.2)	78	131.5 (53.6)	<0.001

**Outcome normalized**		***N*-valid healthy controls**	**Healthy controls Power (%Wmax) [Mean (*SD*)]**	***N*-valid patients**	**Patients Power (%Wmax) [Mean (*SD*)]**	***p*-value**

**VT1_norm**		23	48.6 (9.3)	30	40.7 (11.3)	0.008
**VT2_norm**		23	80.0 (6.8)	29	77.3 (8.7)	0.238
sEMG Th1_norm*						
	Right VL	10	72.7 (9.1)	6	39.9 (18.2)	<0.001
	Left VL	n.a.	n.a.	5	33.6 (19.5)	n.a.
	Right VM	5	70.8 (8.6)	8	46.8 (20.8)	0.033
	Left VM	n.a.	n.a.	6	42.7 (25.2)	n.a.
	Right RF	6	63.8 (21.1)	1	55.7 ()	n.a.^†^
	Right BF	4	65.6 (18.2)	1	67.1 ()	n.a.^†^
	**All muscles**	25	48.6 (9.2)	27	43.0 (20.3)	<0.001
sEMG Th2_norm*						
	Right VL	21	90.8 (5.2)	23	86.8 (8.9)	0.078
	Left VL	n.a.	n.a.	14	84.2 (10.8)	n.a.
	Right VM	10	89.1 (4.8)	18	85.2 (8.6)	0.206
	Left VM	n.a.	n.a.	14	84.5 (8.7)	n.a.
	Right RF	12	84.0 (14.9)	5	80.6 (10.4)	0.655
	Right BF	5	86.8 (13.0)	4	83.6 (9.3)	0.686
	**All muscles**	48	88.3 (9.5)	78	85.0 (9.1)	0.053

*VT1, first ventilatory threshold; VT2, second ventilatory threshold; sEMG Th1, first surface EMG threshold; sEMG Th2, second surface EMG threshold; VT, ventilatory threshold; HC, healthy controls; NMD, neuromuscular disorder; VL, vastus lateralis; VM, vastus medialis; RF, rectus femoris; BF, biceps femoris.*

*sEMG Ths are displayed for all muscles combined. Therefore N-valid of the sEMG Ths exceeds the number of participants included in the study. ^†^p-values were not reported as only one data point for the NMD patients was available, making the comparison between HC and NMD patients not meaningful. Remark: normalized sEMG Th1 and sEMG Th2 values for healthy subjects contained one subject less for right VM and all muscles because Wmax was missing from one participant, and data could not be normalized.

## Discussion

### Main Results

The primary aim of this study was to gain more insight into performance fatigability in patients with NMD by determining the relative timing of both sEMG Ths and the VTs in patients with NMD compared with HC during a maximal ergospirometry cycling exercise test. We expected to find significantly lower normalized values for sEMG Ths and VTs (calculated as %Wmax) in NMD patients than HC. This was, however, only true for sEMG Th1 (*p* < 0.001) and VT1 (*p* = 0.008), and not for VT2 (*p* = 0.238) and sEMG Th2 (*p* = 0.053). In line with our hypothesis, the normalized sEMG Th1 (*p* < 0.001) and sEMG Th2 (*p* = 0.031) occurred at relatively lower power values than the VTs in patients compared to HC. When looking at all muscles combined, the relation between VT2 and sEMG Th2 in NMD patients shows both fixed bias and proportional bias. This indicates that the power at sEMG Th2 was significantly higher than that at VT2. In the study of Smirmaul et al. (2010), non-cyclists showed higher relative power output values for the sEMG Ths than cyclists for the VL muscles. The authors attribute this finding to the lower anaerobic capacity of non-cyclists to keep the specific work of cycling (Smirmaul et al., 2010). Based on physiological measurements, cycling efficiency, and preferred cadence, some studies have suggested differences in muscle recruitment patterns between untrained and highly trained cyclists (Hug and Dorel, 2009). Takaishi et al. (1998) suggested that cyclists have a certain pedaling skill regarding the positive utilization of knee flexors up to the higher cadences, which would contribute to a decrease in peak pedal force and which would alleviate muscle activity for the knee extensors (VL and VM). However, the number of subjects in the current study is too small to compare the timing of sEMG Ths between different leg muscles.

### Feasibility

Most previous studies using sEMG during a maximal ergospirometry cycle test investigated healthy athletes (Ertl et al., 2016). In NMD patients, a maximal cycle test is not always feasible because of muscle weakness. Indeed, three patients could not complete the exercise test because of muscle weakness. Not all data could be collected; the percentage of correct data was higher for the ergospirometry data than the sEMG data. Reasons for the absence of sEMG data were a faulty cable, disturbance in EMG signal, or human error. Identifying sEMG Th1 was more difficult than for the sEMG Th2 because the relative rise in amplitude was less pronounced and, therefore, more difficult to detect visually. In the study of Lucia et al. (1999), there also was a higher detection rate of sEMG Th2 than sEMG Th1. Among all reviewed studies by Ertl et al. (2016), there seems to be a consensus that the first non-linear EMG increase refers to additional recruitment of motor units due to a compensation mechanism based on reduced contractility. A second non-linear increase of the sEMG signal should indicate recruitment of different muscle fiber types (Lucia et al., 1999), to maintain the required energy supply for muscle contraction and compensate for the loss of contractility due to the decreased conductivity as a result of performance fatigability (Lucia et al., 1997; Bearden and Moffatt, 2001; Candotti et al., 2008). The increased contribution of fast-twitch motor units results in a faster fatigue process of the involved muscles because of the increased H^+^ and lactate concentrations, which could explain the difference in the amplitude rise between sEMG Ths. Furthermore, Candotti et al. (2008) reported a correlation between the percentage of fast-twitch muscle fiber recruitment and the lactate accumulation after both sEMG Ths.

### Reliability

The inter-rater reliability for sEMG Th2 was high (>0.95) for all leg muscles. On the contrary, the overall inter-rater reliability for sEMG Th1 was low and only high for the right RF (0.99). The difficult detection of sEMG Th1 could be the most important reason for the low reliability. In determining the reliability, measurements for all muscles were combined. Consequently, the number of EMG measurements was higher than the number of ergospirometry measurements, which potentially biased the inter-rater reliability. The high ICC for sEMG Th2 is not surprising given the relatively small disagreements between raters.

All parameters showed excellent test–retest reliability. However, the absolute values for VT2 were significantly higher (15%) in the retest compared to the test (*p* < 0.001). This could have negatively influenced the correlation between the sEMG Th2 and the VT2. Previous studies have demonstrated that the presence of a competitor improves performance, often based on psychological and emotional responses, such as increasing motivation, positively influencing the balance of willingness to exert the required effort versus negative factors of fatigue and risk (Corbett et al., 2012). In this study, patients were competing with themselves, as they knew the test results of the first attempt before the retest session.

Another possible explanation is that some patients were afraid to cycle to their maximum the first time. They were more confident in the retest. Graef et al. (2008) suggested performing constant workload tests because incremental tests are more dependent on motivational aspects of the participants due to maximal workload. And finally, the difference could be explained by the possible low inter-rater reliability of VT2, which is based on visual assessment. Assessment of VT2 was complicated in NMD patients because of the more considerable variability in the data. The sEMG Th2 showed better test–retest reliability than the VT2 (better absolute agreement between test and retest). The reason for this better reliability is not known yet. Piucco et al. (2020) used a mathematical method to determine the sEMG Ths. The program calculated the regression lines for all possible divisions of the data into 2 (2-lines) and into 3 (3-lines) contiguous groups (Piucco et al., 2020).

### Correlations Between Ventilatory and Surface Electromyography Thresholds and Underlying Pathophysiology

The normalized sEMG Th1 and Th2 occurred at relatively lower power values than the VTs in patients compared to HC, which was most pronounced for the sEMG Th1. Muscle fiber type can influence the timing of the sEMG Ths (Beck et al., 2007). Previous research with muscle biopsy samples showed this pattern: at about 40% of VO_2_max, almost only type I muscle fibers are recruited, whereas at about 60% of VO_2_max (sEMG Th1), both type I and IIa are activated, and during strenuous exercise [about 90% of VO_2_max (sEMG Th2), fibers of type I, IIa, and IIx are recruited (Vollestad and Blom, 1985)]. The difference in timing in the thresholds could have been influenced by the consequences of disuse as NMD patients generally have lower physical activity levels than healthy persons (Jimenez-Moreno et al., 2017). Detraining and disuse reduce the lactate and VTs, especially VT1 (Londeree, 1997). The reversibility of many NMD-related changes in neuromuscular functioning in response to exercise suggest the extent to which these alterations are related to activity status rather than muscle pathology (Ferguson et al., 2016; Heskamp et al., 2020). The performance fatigability profile following muscle disuse atrophy causes a slow to fast fiber transition and could (partially) explain the difference in timing of the sEMG Ths in patients, compared to healthy subjects (Ahmetov et al., 2012).

In contrast to fast-twitch, type II, and slow-twitch, type I fibers are generally much more fatigue resistant. But the difference in timing of the sEMG Ths and VTs between patients and HC cannot be solely explained by the presence of disuse in patients. The difference in timing remained after normalizing for power values. It is known that NMD patients have a different ratio between muscle fiber types compared to healthy people due to the progression of the disease (Glaser and Suzuki, 2018). The majority of the NMD patients in this study were affected by FSHD, which has a wide range of clinical severity, age of onset, and rate of disease progression (Hamel and Tawil, 2018). In FSHD, an impairment in differentiation from slow to fast muscle fibers correlates to the size of the contracted D4Z4 region (Celegato et al., 2006). However, it must be noted that muscles of patients with myotonic dystrophy show a prominent loss of type 1 fibers (Mateos-Aierdi et al., 2015), and mixed atrophy is characteristic for neurogenic disorders (Bongers et al., 2013). Another possible explanation for the difference in the relatively early occurrence of sEMG Th1 in patients compared to HC is that motor units in NMD patients generate less power per unit than HC. Lassche et al. (2021) found that specific force, the amount of force generated per unit of muscle tissue, is reduced in patients with inclusion body myositis, oculopharyngeal muscular dystrophy, and FSHD. Other studies show that specific muscle force is reduced in patients with myotonic dystrophy (Sutcu et al., 2019). In patients with spinal muscular atrophy, a significant increase in the number of type I fibers and no detectable type II fibers are observed (Biral et al., 1989).

### Surface Electromyography Thresholds in Dynamic Conditions

To the best of our knowledge, this is the first study in which signs of performance fatigability during a dynamic exercise test are measured using sEMG Ths in NMD patients. Other studies with EMG in NMD patients used isometric strength exercises (Schulte-Mattler et al., 2003; Schillings et al., 2007; Bachasson et al., 2014; Beretta-Piccoli et al., 2021). When using isometric contractions, problems associated with the signal complexity of dynamic contractions on EMG signals are avoided. However, for NMD patients, it is necessary to measure performance fatigability during dynamic movements and at different loads, as it reflects the situation of patients in daily life. Different changes in EMG parameters are observed during a dynamic contraction compared with a static contraction. In a study comparing changes in sEMG parameters during static and dynamic fatiguing contractions, the sEMG amplitude increased during both the static and dynamic contractions.

On the other hand, muscle fiber conduction velocity significantly decreased during static contractions but did not decrease during dynamic contractions. This discrepancy could be caused by the difference in blood flow, which is maintained during a dynamic contraction and probably removes the metabolic byproducts (Masuda et al., 1999). Most previous research using sEMG during dynamic conditions was conducted in healthy male (elite) cyclists. In elderly males, the relative sEMG Th2 for the VL muscles was significantly earlier than the VT2 during a bicycle ramp exercise which was in contrast to the timing of the sEMG Th2 in NMD patients (Sasaki et al., 2016). This could be explained by the more pronounced muscular atrophy in NMD patients, as described before. On average, half of the subjects in the current study were female. Women differ from men in muscular function; their muscular performance is lower than men’s. To the best of our knowledge, only one study described the timing of the sEMG Th2 in women. In women, the sEMG Th2 occurred at on average 83% of Pmax, compared to 90.8 and 86.8% in the HC and NMD patients in this study, respectively (Long et al., 2017).

In a study using a single treadmill running test, the sEMG Th1 proved to be the most sensitive method for estimating the running pace that can be sustained over a long period without evidence of fatigue in amateur indoor soccer players, and it could differentiate fatigue thresholds between lower extremity muscles. The authors question the utility of the sEMG Th2 in treadmill running as they could not identify this threshold in nearly 50% of subjects (Crozara et al., 2015).

### Limitations

There are some limitations to this study. First of all, this was an exploratory study with a small sample size. A larger sample size might have been better in detecting significant results for comparing the protocols and groups. However, it was still possible to obtain slope incline and decline effects with the current sample size during different loads. In addition, different muscles were measured in various participants. As a result, some muscles are measured more frequently than others, which might have influenced the results, especially regarding the variability of the results.

As Vigotsky et al. (2017) concluded in their review on interpreting signal amplitudes in sEMG studies in sport and rehabilitation, sEMG is a valuable tool for gaining insight into the neuromuscular system, musculoskeletal modeling, and basic science work. Still, its practical applicability is limited at present. Important mechanistic details of sEMG, such as signals being confounded by peripheral factors and data not representative of a muscle, must be considered when attempting to draw conclusions (Vigotsky et al., 2017). Measurement of sEMG signs of performance fatigability during vigorous exercise is more complicated than isometric exercise because of the inherently more significant number of extraneous variables in contend with, e.g., variable muscle length and cross-sectional area, movement of the muscle below the electrode, etc. (Hof, 1997). However, as cycling is a cyclic and constant movement, we expect that the influence of these variables on the sEMG signal is limited.

During the measurements, motion artifacts were prevented by carefully fixing the wires and checking for artifacts in the real-time signal. Using a bandpass filter of 20–450 Hz eliminated low-frequency noise caused by movement artifacts. Several subjects changed their sitting position on the bike at the end of the measurement. Savelberg et al. (2003) showed that the length at which muscle activity occurred was influenced by trunk angle in the BF and RF. In addition, changing position also caused temporary changes in sEMG activity, which made accurate threshold determination more difficult. Ertl et al. (2016) describe that the determination of the sEMG Ths was consistent in the VL, VM, and RF muscles. sEMG signals of other, smaller leg muscles did not show sEMG Ths as consistently.

The NMD patients, mean age 55.0 (30.1–75.0) years, were significantly older than the HC, which were not age-matched mean age 36.4 (19.1–59.6) years, *p*-value < 0.001. Aging is associated with muscle fiber-type shifting, leading to a more significant proportion in fatigue-resistant type I muscle fibers (Callahan et al., 2016). Consequently, active skeletal muscles of older adults may experience less metabolic perturbation in hydrogen ions and inorganic phosphates during isometric fatiguing contractions and, therefore, less fatigability (Deschenes, 2004).

### Future

Most studies with sEMG during exercise have been performed in the laboratory and thus have used stationary cycle ergometers. Exhaustive tests, like this incremental cycling test, are ecologically invalid, i.e., not relevant for activities in daily life. Second, it is advised to develop a more reliable method to detect sEMG Th1, e.g., artificial intelligence, as this threshold could be valuable for daily life measurements. Different sEMG Th detection methods have been used, such as visual and multi-segment linear regression methods (Ertl et al., 2016). The mathematical detection method of sEMG Ths is more objective. Still, no method exists with the complex pattern recognition skill required to recognize and reject traces in which the sEMG Th is confused by artifacts.

To end, because fatigue is experienced as a multi-dimensional symptom with short-term or long-term duration, relying on one outcome measure alone can be difficult. Future studies should include performance fatigability and perceived fatigability measures to correlate objective and subjective fatigue in their subjects/patients and include age-matched HC. Further research is needed to address a multi-model approach to measure performance fatigability in NMD to differentiate performance fatigability developed from the disease itself compared to normal physiological and aging processes, understand the concomitant nature of performance fatigability and perceived effort, as well, identify appropriate therapeutic interventions to minimize performance fatigability in patients with NMD. Preferably, in future research, more homogeneous patient groups should be included to determine the influence of the specific pathology.

## Data Availability Statement

The raw data supporting the conclusions of this article will be made available by the authors, without undue reservation, to any qualified researcher.

## Ethics Statement

The studies involving human participants were reviewed and approved by Medical Ethics Review Committee Radboud University Medical Center Nijmegen, Netherlands. The participants provided their written informed consent to participate in this study.

## Author Contributions

NV and MJ, the principal authors of the manuscript, conceived, designed and supervised the experiments. CS, DT, VB, and DS contributed to discussing the results and the writing and editing of the manuscript. MJ analyzed the data. NV obtained ethical approval. All authors read and approved the manuscript.

## Conflict of Interest

The authors declare that the research was conducted in the absence of any commercial or financial relationships that could be construed as a potential conflict of interest.

## Publisher’s Note

All claims expressed in this article are solely those of the authors and do not necessarily represent those of their affiliated organizations, or those of the publisher, the editors and the reviewers. Any product that may be evaluated in this article, or claim that may be made by its manufacturer, is not guaranteed or endorsed by the publisher.
